# Supplementary material for: Perceived barriers in accessing sexual and reproductive health services for youth in Lao People’s Democratic Republic
Source: PLoS One. 2019 Oct 29;14(10):e0218296. doi: 10.1371/journal.pone.0218296 (PMC6818758; doi:10.1371/journal.pone.0218296)

**Coding scheme**

Coding scheme current situation
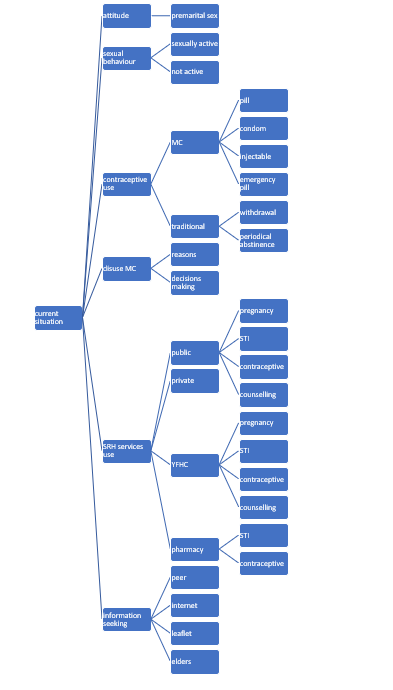


Coding scheme barriers


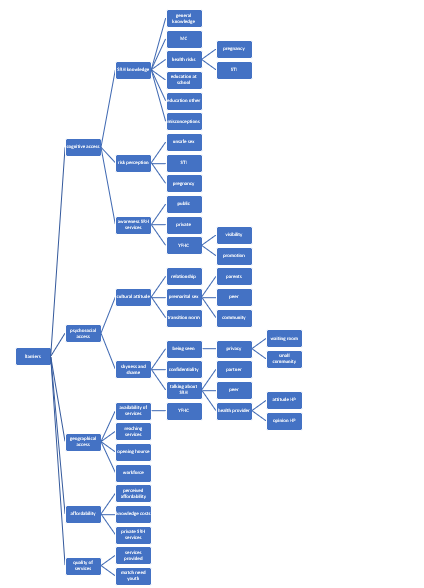


Coding scheme solutions


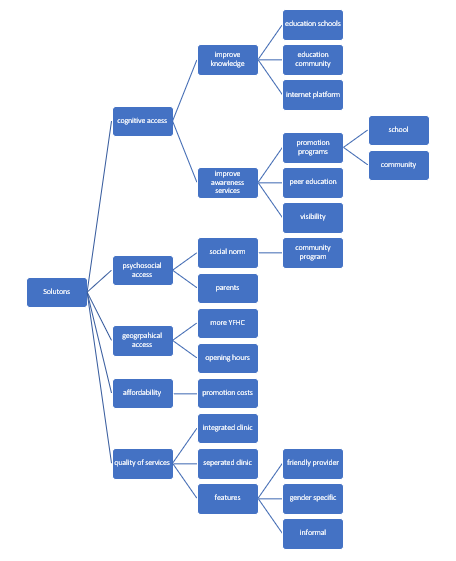

Supplement: S1 File — (DOCX) [file pone.0218296.s001.docx]
