# Supplementary material for: Perceived barriers in accessing sexual and reproductive health services for youth in Lao People’s Democratic Republic
Source: PLoS One. 2019 Oct 29;14(10):e0218296. doi: 10.1371/journal.pone.0218296 (PMC6818758; doi:10.1371/journal.pone.0218296)
Supplement: Attachment — Submitted filename: PONE-D-19-14849.docx [file pone.0218296.s002.docx]

PONE-D-19-14849

The article is well written and addresses an important public health issue. The authors assessed perceptions of barriers to youth friendly services. The article should be accepted with the following minor revisions:

Introduction

- The acronym YFHC is spelt out in two ways (youth-friendly health centres/ only two Youth Friendly Health Clinic). The authors should be consistent.
- The objective of the study is stated as “to explore their perceptions regarding accessibility of SRH services for youth” while the title focussed on “barriers”. The word barriers is more generally and could be a better term to use here and the rest of the article.

Methods

The first line of the section on study tools cites table 1 as the study tool yet table 1 in the document actually is a description of study participants. This error should be corrected.

“A semi-structured interview guide was developed for each participant group (Table 1).”

The terms “*cognitive accessibility”, “psychosocial accessibility”, “geographical accessibility”, ‘affordability”* and *“quality of services”* should be defined.

The vignettes used in the study could be made available for readers.

Ethical approval – This study recruited youth who were “under the age of majority”. It is not clear if this inclusion of these under age participants without parental consent was approved by the ethical review committee.

Results

The authors report that “The majority of adolescents did not report fearing negative attitudes of health providers in public SRH services. Even though health providers are from an older generation, adolescents assumed that they were professionals who would not treat adolescents in a negative way” yet later report that the adolescents were concerned about lack of confidentiality. It is possible to assume that this perceived lack of confidentiality could be related to the age of the health care providers. The health care providers may find it easy to report them to their parents since they were of the same generation and shared views on adolescent sexuality. The authors later acknowledge that this is the reason why 4 adolescents sought SRH services outside their own district.

Quality of services are ruled out as being of concern to the youths. Privacy and confidentiality are important dimensions of quality. This could be clearer if the authors defined what the elements of quality youth friendly services are.

Discussion

“Youth-friendly services are available in Vientiane and Savannakhet; the main barrier in accessing YFHCs was being unaware of their existence.” - this statement needs to be qualified – there are only two YFHCs. The accuracy of this statement can only be assessed if information on the geographical size and population of the two services is presented.

The authors rule out the role of quality of youth friendly services as being a barrier yet acknowledged in the results the concerns of privacy and confidentiality which could actually be elements of quality. The authors should reconsider this statement.

The role of lack of privacy and confidentiality has not been adequately discussed. This could be a key barrier to YFHC services.

Recommendations

There are recommendations about provision of youth friendly services in schools which is not adequately supported by the data presented.
