# Supplementary material for: Perceived barriers in accessing sexual and reproductive health services for youth in Lao People’s Democratic Republic
Source: PLoS One. 2019 Oct 29;14(10):e0218296. doi: 10.1371/journal.pone.0218296 (PMC6818758; doi:10.1371/journal.pone.0218296)
Supplement: Attachment — Submitted filename: Response to Reviewers.docx [file pone.0218296.s003.docx]

Reviewer 1

| **Comment** | **Answer** |
| --- | --- |
| **General** |  |
| There are a number of grammatical errors across the entire paper. This needs to be addressed | Thank you for your comment. We have had a native English researcher and editor do a thorough check of the text. |
| **Introduction** |  |
| It would have been valuable for the investigators to give the readers a global and an Asia perceptive on assess to sexual and reproductive health care services before focusing on the situation in Lao PDR. Systematic reviews could be useful for this… This would help the readers gain better insight on the topic of focus. | Many thanks for your comment; we have added a paragraph to the introduction using reviews from Asia. |
| **Method** |  |
| The methods section does not flow well. There is need to better organize the methods section to enhance flow of content. The investigators could consider the following flow: Study design, Study site, Study population, Sample size and sampling technique, Data collection tool, Data collection procedure (including ethical consideration, recruitment, data collection processes) and Data analysis. While doing this, it is important to provide relevant content for each section. As it is, the study design starts with the information that would be best placed in the study population section. | Many thanks for your comment, we have adapted the flow of the methods section as suggested. Track changes show where text was revised. |
| In the study tools, the investigators mention that the interview guides were based on concepts of the theoretical model yet there is not reference to which theoretical model being applied… Which theoretical model is this? What are the constructs? Are you adapting or adopting all the constructs of the model?? | We adapted the definitions and conceptual frameworks of Bertrand, Hardee, Magnani, & Angle (1995), Hardee et al., 2014, Levesque et al. (2013) and the United Nations Committee on Economics, Social, and Cultural Rights (UNCESR). The merged conceptual model consists of five key concepts: *cognitive accessibility*- the extent to which youth are aware of the existence of SRH services and their perceived SRH needs, including knowledge regarding MC and risks for pregnancies and STIs when having sexual intercourse; *Psychosocial accessibility*- the extent to which cultural and social norms influence youth to use SRH services and their perceived agency and autonomy to make decisions by themselves; *geographic accessibility*- the extent to which SRH services can be reached physically and the amount of time it takes; *affordability*- the perceived agency of youth to pay for reaching and using SRH services; and finally, *Quality of services*- the extent to which the provided SRH services match with the perceived needs of youth: accessible, acceptable and appropriate (WHO, 2015) |
| In the abstract there is mention of semi-structured interview guides being used in the study. However in the study tools section of the manuscript there is no mention of structured questions…. All quest | When we write ‘semi-structured interview guide’ we mean that the interview is structured for content and sequence of questions but the questions themselves were mostly open. |
| In the location section, it would have been useful to provide statistics on the 2 study sites (Vientiane and Savannakhet)… What is the population of adolescents in the 2 study sites? What are the adolescents sexual debut/pregnancy rates in the 2 regions? Given the focus on the study this information would help the readers understand the 2 study sites better and provide an even stronger justification for including the 2 sites. | The provinces were selected because of the availability of the YFHC. We have added to the text on location the basic statistics related to adolescent RH in the two /provinces where the study was done. |
| In the sample and sampling technique the investigators do not state how they came up with the target numbers of 22 young people and 7 health providers. The aim of qualitative studies is to achieve saturation. Was this achieved? It was also interesting that the investigators did not stratify the sample based on having accessed SRH services. This would be an important strata for this study prior to recruitment… As it is there were only 4 out of 18 young participants who had no SRH experience….Hence the main question is whether the 4 young persons were adequate to achieve saturation for this category of participants…. | Thank you for your question and comment. We have added text in the methods section to explain the sample sizes in the methods section.  We have also added text on this point to the section on limitations in the discussion. |
| It would have also be important to provide information about the interviewers…. What qualifications did they have? Were they trained prior to data collection? | Two native Lao speakers trained in qualitative research methods (the first and fourth authors) conducted all interviews in Lao from the same semi-structured interview guide, which included open-ended questions and probing questions. This is now clarified in the Methods section. |
| In the ethical approval the investigator state that they had 3 participants below 18 years and that these participants felt that they were able to give consent…. What does this mean? What are the laws in Lao regarding engaging minors in studies? Were this emancipated minors | This study was part of the project “Unmet need for family planning: New approach model, Lao PDR”.   - First of all, parental consent was not required, however village health workers did inform parents when selecting youth for interviews and asked for their approval. - The IRB did approve of this research. In our case respondents below the age of 15 required signed consent of parents. Participants between 15-18 did not require parental consent. The IRB in Laos does not require parental consent for non-invasive research with participants 15 or older. We did submit a consent form to the IRB in case we would have participants below the age of 15. This form needed to be signed by parents. However, in the current study we did not include youth below 15. We have attached the letter of approval of the ethical committee. |
| **Result** |  |
| Table 1 could be presented better. A simple descriptive table with the median age of participants, level of education, sexual encounter and accessed SRH services would have sufficed. | Many thanks for your comment. We have added an overview table as you described but still kept the original table (now Table 2) as it provides additional information that we feel is relevant. |
| There is need to organize the themes within in the results section better. For example the first theme should be uptake of SRH services. Under this theme subthemes should be, perception about sexual debut, access to SRH, conceptive use, and SRH information channels. There second theme should be barriers to SRH services with focus on barriers only | Although we see merit in the alternative description of themes, after careful deliberation we have decided to describe the themes as we had done. First the behavior of adolescents is described, preceded by information about participants; second their views on access following the theoretical perspective we applied. We have added more information on the theoretical perspective in the methods. We believe that this together provides coherence to the results and the paper as a whole. |
| Under the sub-theme SRH services the investigators highlight health facility barriers i.e lack or privacy, gender of the provider. Yet the investigators did not consider these are barriers to SRH services. I would therefore recommend that these be moved to barriers | Indeed we briefly touch upon some of the barriers earlier to improve understanding of the current use of services. The barriers are further unraveled later: e.g. the privacy is further described largely under psychosocial accessibility.  The gender of the provider was included in earlier versions and then was cut from the barriers section. Based on your suggestion We have added this again under quality of services. |
| It is surprising the even under the theme barriers to access of SRH services, there is mention of positive aspects. For example, the investigators state that the young people did not report negative provider attitudes, cost related issues, geographical accessibility, and aspects of quality of services…. Given that the theme focuses on barriers, why are the investigators mentioning these positive aspects?? This is a bit confusing. | - There were contrasts of perspective on barriers to access between providers and adolescents. Providers still stated that provider attitudes, cost related issues, geographical accessibility, and aspects of quality of services remain barriers to access, while the adolescents themselves did not report that provider attitudes, cost related issues, geographical accessibility, and aspects of quality of services were barriers for them. The only barrier that adolescents mentioned was cognitive accessibility. Nonetheless, provider attitudes, cost related issues, geographical accessibility, and aspects of quality of services could still be barriers in some places; as one or two adolescent mentioned that a Adolescents living in the rural district Songkhone were an exception, indicating that their district hospital was hard to reach due to poor infrastructure. Therefore, they preferred to obtain their SRH services in Thailand, making it clear that some geographical accessibility barriers remain. |
| Also confusing is that under geographical accessibility there is mention that it was not perceived as a barrier. Then later the investigators state that it was a barrier among the rural population. Which is which? It was also surprising that under geographical accessibility they mention insufficient services. Insufficient services is not the same as geographical accessibility. Insufficient services means that the facilities are available but they are not providing adequate services or the services are not tailored to the needs of the target population. | Thank you for your comments. We have revised accordingly:  - The text on the limited number of service site has been moved to ‘availability’.  - The only geographical barrier was the distance of travel to the service site. As adolescents mentioned that they would go across the border to Thailand for service rather than going to the youth clinic which was too far. In this case, we do not know whether the cross-border service is youth-friendly. |
| I wish the investigators applied the socio-ecological model to explore the barriers to adolescent accessing SRH services. This would have provided a more comprehensive perceptive of barriers from the intrapersonal, interpersonal, community, facility, and policy levels | Thank you for your suggestion. We are working on an upcoming article in which we do apply the social ecological model to the situation of teenage pregnancy and access to care in Lao PDR, for which we have data from range of stakeholders. |

Reviewer 2

| **Comment** | **Answer** |
| --- | --- |
| **General** |  |
| The article is well written and addresses an important public health issue. The authors assessed perceptions of barriers to youth friendly services. The article should be accepted with the following minor revisions: | Many thanks for your kind remarks. |
| **Introduction** |  |
| The acronym YFHC is spelt out in two ways (youth-friendly health centres/ only two Youth Friendly Health Clinic). The authors should be consistent. | Thank you for your comment, we have now consistently used Clinic. |
| The objective of the study is stated as “to explore their perceptions regarding accessibility of SRH services for youth” while the title focused on “barriers”. The word barriers is more generally and could be a better term to use here and the rest of the article. | Many thanks for your comment, we have improved the text, which now reads: “Perceptions of barriers to accessibility of SRH services for youth*,* to stimulate evidence-informed decision-making in health policy formulation and implementation” |
| **Method** |  |
| The first line of the section on study tools cites table 1 as the study tool yet table 1 in the document actually is a description of study participants. This error should be corrected. “A semi-structured interview guide was developed for each participant group (Table 1).” | Thank you for your comment, we have removed this incorrect reference to table 1. |
| The terms “cognitive accessibility”, “psychosocial accessibility”, “geographical accessibility”, ‘affordability” and “quality of services” should be defined. | Many thanks for your comment, we have added text to the introduction and methods sections explaining the terms used and where they came from. |
| The vignettes used in the study could be made available for readers. | Thank you for your suggestion; we will attach the English version of vignettes and interview guide as supplements. |
| Ethical approval – This study recruited youth who were “under the age of majority”. It is not clear if this inclusion of these under age participants without parental consent was approved by the ethical review committee. | Including adolescents under the age of majority without parental consent was part of the proposal approved by the Ethics Committee for Health Research of the University of Health Sciences, Lao PDR. Parental consent in Lao PDR is only required for children under 15 years of age. |
| **Result** |  |
| The authors report that “The majority of adolescents did not report fearing negative attitudes of health providers in public SRH services. Even though health providers are from an older generation, adolescents assumed that they were professionals who would not treat adolescents in a negative way” yet later report that the adolescents were concerned about lack of confidentiality. It is possible to assume that this perceived lack of confidentiality could be related to the age of the health care providers. The health care providers may find it easy to report them to their parents since they were of the same generation and shared views on adolescent sexuality. The authors later acknowledge that this is the reason why 4 adolescents sought SRH services outside their own district. | Thank you for this comment; it is a good point. During the interviews the youth probably responded in relation to their actual interaction with the health care provider. They did not associate that with the fear of a later problem from gossip or reporting to parents. |
| Quality of services are ruled out as being of concern to the youths. Privacy and confidentiality are important dimensions of quality. This could be clearer if the authors defined what the elements of quality youth friendly services are. | Many thanks for your comment, we have tried to make this clearer in the text and have added a paragraph in the discussion. |
| **Discussion** |  |
| “Youth-friendly services are available in Vientiane and Savannakhet; the main barrier in accessing YFHCs was being unaware of their existence.” - this statement needs to be qualified – there are only two YFHCs. The accuracy of this statement can only be assessed if information on the geographical size and population of the two services is presented. | Many thanks for your comment. We have added information on the geographical size and population of the two study sites to the Methods section. |
| The authors rule out the role of quality of youth friendly services as being a barrier yet acknowledged in the results the concerns of privacy and confidentiality which could actually be elements of quality. The authors should reconsider this statement. | Many thanks for your comment, we have tried to clarify this point in the Discussion text, and have added a paragraph there. |
| The role of lack of privacy and confidentiality has not been adequately discussed. This could be a key barrier to YFHC services. | As mentioned above, the adolescents did not appear to relate these issues to the questions about quality of service and barriers they perceived. However we have included them as potential barriers in the Discussion section. |
| **Recommendations** |  |
| There are recommendations about provision of youth friendly services in schools which is not adequately supported by the data present | Many thanks for your comment. We have revised the text to make clear that the suggestions came from the data collected, not from the authors |
